# Sex-Specific Associations Between Hyperuricemia and Kidney Stone Disease in a Large Taiwanese Cohort

**DOI:** 10.7150/ijms.109742

**Published:** 2026-01-01

**Authors:** Meng-Shiang Liu, Shuo-Hung Wang, Jia-In Lee, Szu-Chia Chen, Shu-Pin Huang, Jiun-Hung Geng

**Affiliations:** 1 Department of Post-Baccalaureate Medicine, College of Medicine, Kaohsiung Medical University, Kaohsiung 807378, Taiwan.; 2 Department of Psychiatry, Kaohsiung Medical University Hospital, Kaohsiung Medical University, Kaohsiung 807378, Taiwan.; 3 Department of Internal Medicine, Kaohsiung Municipal Siaogang Hospital, Kaohsiung Medical University, Kaohsiung 812015, Taiwan.; 4 Department of Internal Medicine, Division of Nephrology, Kaohsiung Medical University Hospital, Kaohsiung Medical University, Kaohsiung 807378, Taiwan.; 5 Faculty of Medicine, College of Medicine, Kaohsiung Medical University, Kaohsiung 807378, Taiwan.; 6 Research Center for Environmental Medicine, Kaohsiung Medical University, Kaohsiung 807378, Taiwan.; 7 Graduate Institute of Clinical Medicine, College of Medicine, Kaohsiung Medical University, Kaohsiung 807378, Taiwan.; 8 Department of Urology, Kaohsiung Medical University Hospital, Kaohsiung Medical University, Kaohsiung 807378, Taiwan.; 9 Department of Urology, School of Medicine, College of Medicine, Kaohsiung Medical University 807378, Kaohsiung, Taiwan.; 10 Institute of Medical Science and Technology, College of Medicine, National Sun Yat-Sen University, Kaohsiung 804201, Taiwan.; 11 Department of Urology, Kaohsiung Municipal Siaogang Hospital, Kaohsiung 812015, Taiwan.

## Abstract

**Background:** Kidney stone disease (KSD) is a growing global health issue, while hyperuricemia has been linked to various health conditions. This study aimed to explore the association between hyperuricemia and KSD.

**Methods:** This study analyzed the data of 118,963 generally healthy participants aged 30-70 years from the Taiwan Biobank. KSD was defined based on self-reported diagnoses, and hyperuricemia was defined as a serum uric acid level > 7 mg/dL in men and > 6 mg/dL in women. Key variables including age, sex, body mass index, lifestyle factors, presence of hypertension, diabetes, and chronic kidney disease were recorded. Statistical analysis was conducted using descriptive statistics and logistic regression to evaluate the association between hyperuricemia and KSD. Odds ratios (ORs) and corresponding 95% confidence intervals (CIs) were reported, with statistical significance set at p < 0.05. An additional causal mediation analysis was performed to assess the mediating role of gout.

**Results:** Among the 118,963 participants, 23,094 (19.4%) had hyperuricemia. Those with hyperuricemia were older, had a higher body mass index, and were more likely to smoke, consume alcohol, and have elevated blood pressure. They also had higher rates of comorbidities such as hypertension, diabetes, metabolic syndrome, chronic kidney disease, and gout, along with worse laboratory profiles. Multivariable analysis revealed that hyperuricemia was significantly associated with an increased risk of KSD (OR: 1.155, 95% CI: 1.089-1.224, p < 0.001). When stratified into four exposure groups, the prevalence of KSD was 5.4% in participants without hyperuricemia or gout, 8.5% in those with hyperuricemia alone, 17.0% in those with gout alone, and 15.7% in those with both conditions; the adjusted ORs were 1.178, 1.522, and 1.505, respectively, compared with the reference group. The risk was more pronounced in females, particularly those in higher uric acid quartiles, whereas in males, the association was weaker and became nonsignificant after full adjustments. Mediation analysis further indicated that gout explained approximately 24% of the association between hyperuricemia and KSD, while the direct effect of hyperuricemia on KSD remained significant.

**Conclusion:** Hyperuricemia was an independent risk factor for KSD in this large Taiwanese cohort study, with a stronger association observed in females. Gout partially mediates this relationship, suggesting overlapping yet distinct pathways linking hyperuricemia to kidney stone formation. These findings underscore the importance of sex-specific management strategies for KSD. Routine monitoring of serum uric acid levels is recommended to mitigate KSD risk, especially among high-risk individuals. Future research should focus on elucidating the long-term impact of hyperuricemia on KSD and tailoring prevention strategies to regional and population-specific needs.

## Introduction

Kidney stones represent a significant global health issue and negatively affect urinary health. The incidence rates are increasing worldwide, and particularly in specific regions and populations. For example, ultrasound surveys reported a prevalence of 6.4% in China 1, while there was a steady increase from 2007 to 2018 in the US 2. In Taiwan, kidney stone cases are increasing, influenced by lifestyle factors such as obesity and metabolic syndrome 3-5. Importantly, kidney stones are associated with health risks including acute pain, infection, and chronic kidney disease (CKD), underscoring the urgent need for effective prevention and treatment strategies.

The development of kidney stone disease (KSD) is influenced by several risk and protective factors. Key risk factors include lifestyle choices such as smoking, alcohol consumption, and physical inactivity^6^. Obesity and metabolic syndrome also significantly increase the risk of stone formation, with a strong correlation between higher body mass index (BMI) and the development of KSD 3,5. Although men have historically exhibited a higher prevalence of kidney stones, recent epidemiologic evidence indicates a narrowing sex gap in KSD risk, particularly in younger and postmenopausal women 7. These changes are thought to reflect shifts in metabolic health, hormone levels, and lifestyle factors. Genetic predispositions, particularly in calcium and vitamin D metabolism, have also been shown to increase the susceptibility to kidney stones 8. Conversely, maintaining a healthy weight, engaging in regular physical activity, and ensuring adequate hydration have been shown to be protective factors, reducing the likelihood of stone formation 9,10. Dietary measures such as limiting sodium and oxalate-rich foods have also been shown to help prevent kidney stones 11,12.

Kidney stone composition also differs markedly across age, sex, and metabolic profiles. Calcium oxalate stones are the most common overall, particularly in younger to middle-aged men. In contrast, uric acid stones are more prevalent in older individuals, especially men with metabolic syndrome or type 2 diabetes, due to low urinary pH and insulin resistance 13. Brushite stones, a rare and aggressive subtype of calcium phosphate stones, disproportionately affect younger men with elevated urinary pH and low citrate levels, even in the absence of metabolic syndrome 14. Meanwhile, recent epidemiologic trends have revealed a growing prevalence of kidney stones among women, particularly during reproductive and postmenopausal periods. This is likely driven by increasing rates of obesity, hypertension, and insulin resistance, which are known to favor uric acid and calcium phosphate stone formation 15,16. These demographic and metabolic differences underscore the importance of considering sex-specific factors when evaluating KSD risk.

Chronic hyperuricemia is a well-established risk factor for gout and has also been linked to several metabolic and cardiovascular conditions 17-19. Elevated uric acid levels can lead to monosodium urate crystal deposition, triggering painful gout flares and joint inflammation 20. Beyond its role in gout, hyperuricemia has been increasingly recognized as a marker of broader metabolic dysfunction. While the causal relationships remain under investigation, its associations with inflammatory processes, oxidative stress, and endothelial dysfunction suggest a potential role in various disease pathways 17-20. These systemic effects underscore the clinical importance of monitoring and managing serum uric acid levels in individuals at risk for urate-related complications.

Hyperuricemia is also a key factor in the development of kidney stones, particularly through the crystallization of uric acid in the urinary tract. Elevated serum uric acid levels are associated with increased urinary uric acid excretion, which, when combined with low urinary pH, leads to uric acid stone formation 20. In addition to the formation of urate crystals, hyperuricemia has been shown to contribute to kidney stone formation due to its pro-inflammatory effects 21. The accumulation of urate crystals in the renal tubules can induce inflammation and oxidative stress, further impairing kidney function 22. Mendelian randomization studies have identified a causal relationship between serum uric acid levels and urolithiasis, and shown the increased susceptibility of individuals with genetic predispositions 20. These findings underscore the importance of managing hyperuricemia through lifestyle changes and pharmacological interventions, including urate-lowering therapies, to prevent the development of kidney stones and protect renal function.

Importantly, sex-based physiological differences may influence the relationship between hyperuricemia and the risk of kidney stone formation. However, large-scale studies investigating the sex-specific impact of hyperuricemia in this context remain limited. Therefore, this study aimed to examine their association in a large Taiwanese cohort, with particular emphasis on sex differences in risk and susceptibility.

## Materials and Methods

### Study population

**Figure 1:**
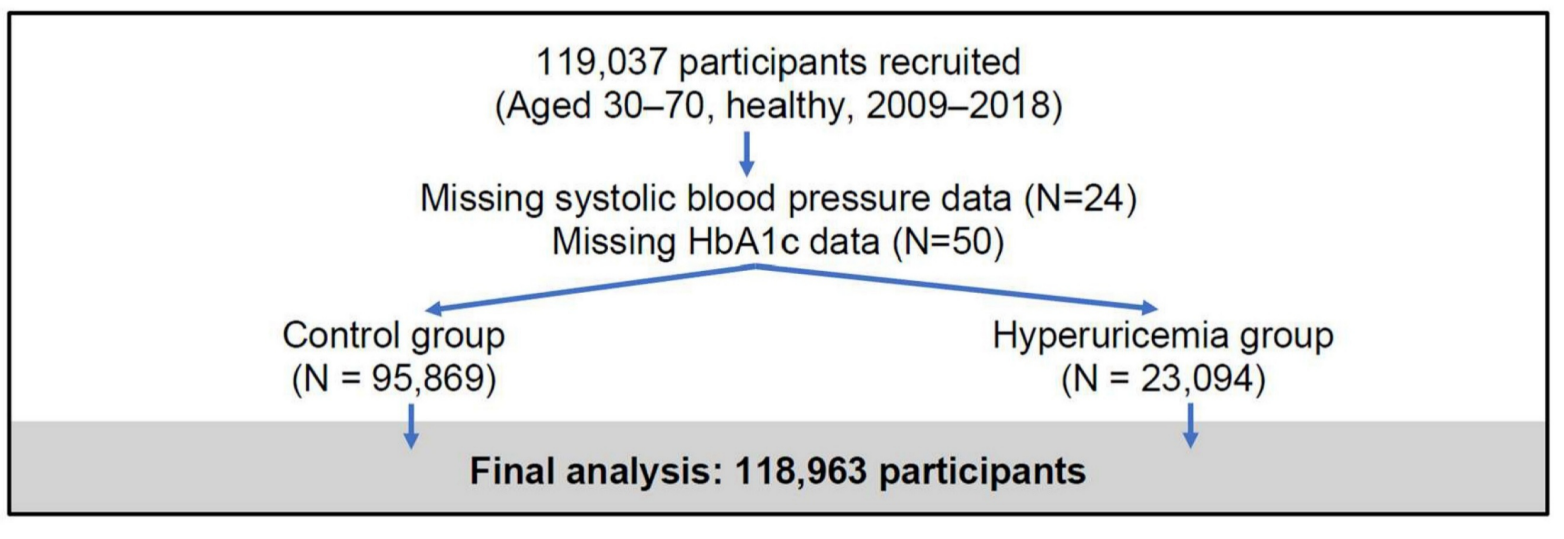
Flowchart of participant recruitment and study inclusion

This study used data from the Taiwan Biobank (TWB)^23^, a large-scale, population-based cohort established to investigate genetic, environmental, and lifestyle factors influencing the health of Taiwanese adults. The TWB recruited generally healthy individuals aged 30 to 70 years from 29 regional collection sites between 2009 and 2018, focusing on participants without major chronic diseases (e.g., cancer, diabetes, cardiovascular diseases). At baseline, comprehensive data were collected, including biological samples (blood, urine, saliva), physical measurements (e.g., height, weight, blood pressure), and detailed health questionnaires covering lifestyle habits, medical history, and family health background. Standardized protocols ensured high data quality, with key biomarkers such as HbA1c and lipid profiles measured in laboratory tests. For this analysis, a total of 119,037 participants were initially enrolled, with 74 individuals excluded due to missing data on systolic blood pressure or HbA1c, resulting in a final sample of 118,963 participants **(Figure 1)**. The TWB dataset provides a robust platform for exploring the complex interplay between genetic predisposition and environmental exposure, supporting studies on disease risk factors and precision medicine initiatives in Taiwan.

### Disease definitions

The presence of KSD was defined based on self-reported diagnoses. Hyperuricemia was defined as an elevated serum uric acid level of > 7 mg/dL (420 μmol/L) in men and > 6 mg/dL (360 μmol/L) in women 24.

### Variable selection

The key variables included age, sex, BMI, lifestyle factors, and clinical markers. Age and sex were included due to their known influence on stone formation, with older individuals and males having a higher risk 25. BMI was included due to its known link with KSD risk 26,27. Lifestyle factors including smoking, alcohol consumption, and physical activity were also recorded, as they are known to affect metabolic health. Hypertension, diabetes, CKD, and gout, were included due to their established associations with kidney stones 28. Related biochemical markers including fasting glucose, estimated glomerular filtration rate (eGFR), and lipid profiles, were also examined.

### Statistical analysis

Descriptive statistics were used to summarize the baseline characteristics of the study participants. Continuous variables were presented as means with standard deviations, while categorical variables were expressed as frequencies and percentages. Group differences between participants with and without hyperuricemia were evaluated using independent t-tests for continuous variables and chi-square tests for categorical variables. To assess the association between hyperuricemia and KSD, univariate binary logistic regression was performed, and odds ratios (ORs) and 95% confidence intervals (CIs) were calculated for each variable. Variables found to be significant in the univariate analysis were subsequently included in a multivariate binary logistic regression model to identify independent predictors of KSD. To examine the dose-response relationship between serum uric acid levels and KSD, the participants were stratified into quartiles based on their serum uric acid concentration. Logistic regression models were applied, with the first quartile serving as the reference group. In addition, to evaluate whether gout mediates the relationship between hyperuricemia and KSD, causal mediation analysis was performed using the nonparametric bootstrap method with 1,000 simulations 28. The total effect, direct effect, indirect (mediated) effect, and the proportion mediated were estimated, adjusting for the same covariates as in the multivariate logistic regression model. Statistical significance was set at p < 0.05. All analyses were conducted using SPSS version 26 (IBM Corp., Armonk, NY, USA) and R version 4.5.1 (R Foundation for Statistical Computing, Vienna, Austria).

## Results

Of the 118,963 participants, 23,094 had hyperuricemia and 95,869 did not **(Table 1)**. Significant differences were observed across various demographic, lifestyle, and clinical characteristics. The participants with hyperuricemia were older (51 ± 11 vs. 50 ± 11 years, p < 0.001), had a higher BMI (26.50 ± 3.93 vs. 23.67 ± 3.53 kg/m², p < 0.001), and were more likely to smoke (37.7% vs. 24.8%, p < 0.001), consume alcohol (13.9% vs. 7.2%, p < 0.001), and have elevated systolic and diastolic blood pressure (127.15 ± 18.46 vs. 118.93 ± 18.36 and 78.38 ± 11.47 vs. 72.74 ± 11.11 mmHg, p < 0.001). Clinically, participants with hyperuricemia also had a higher prevalence of hypertension (20.9% vs. 10.2%, p < 0.001), diabetes mellitus (6.3% vs. 4.9%, p < 0.001), metabolic syndrome (48.6% vs. 25.1%, p < 0.001), CKD (4.9% vs. 0.8%, p < 0.001), gout (12.5% vs. 1.7%, p < 0.001), and KSD (9.4% vs. 5.6%, p < 0.001). In addition, the laboratory profiles of the participants with hyperuricemia were worse than those without hyperuricemia, with higher levels of HbA1c, creatinine, blood urea nitrogen (BUN), and uric acid (all p < 0.001).

Univariate binary logistic regression analysis demonstrated that hyperuricemia was significantly associated with an increased risk of kidney stones (OR: 1.744, 95% CI: 1.656-1.837, p < 0.001) **(Table 2)**. Other significant predictors of kidney stone formation included older age (OR: 1.036 per year, 95% CI: 1.034-1.039, p < 0.001), female sex (OR: 2.914, 95% CI: 2.778-3.057, p < 0.001), higher BMI (OR: 1.075, 95% CI: 1.069-1.081, p < 0.001), smoking (OR: 1.973, 95% CI: 1.881-2.069, p < 0.001), and alcohol consumption (OR: 1.722, 95% CI: 1.606-1.847, p < 0.001). Clinical conditions such as hypertension, diabetes mellitus, metabolic syndrome, CKD, and gout also emerged as significant risk factors, with ORs all exceeding 2.0 (all p < 0.001). Laboratory markers including higher HbA1c, BUN, creatinine, and uric acid levels were also associated with a higher odds of kidney stone formation.

Multivariate logistic regression analysis, adjusting for potential confounders, confirmed that hyperuricemia was an independent risk factor for kidney stone formation (OR: 1.155, 95% CI: 1.089-1.224, p < 0.001)** (Table 3)**. Significant predictors included older age (OR: 1.030, 95% CI: 1.027-1.033, p < 0.001), female sex (OR: 2.332, 95% CI: 2.190-2.483, p < 0.001), higher BMI (OR: 1.028, 95% CI: 1.020-1.035, p < 0.001), smoking (OR: 1.118, 95% CI: 1.055-1.186, p < 0.001), hypertension (OR: 1.611, 95% CI: 1.511-1.717, p < 0.001), diabetes (OR: 1.214, 95% CI: 1.098-1.342, p < 0.001), metabolic syndrome (OR: 1.091, 95% CI: 1.026-1.161, p = 0.005), CKD (OR: 1.209, 95% CI: 1.038-1.407, p = 0.015), and gout (OR: 1.370, 95% CI: 1.253-1.499, p < 0.001). Non-significant associations included alcohol use (OR: 0.940, p = 0.113), physical activity (OR: 0.961, p = 0.124), HbA1c (OR: 0.989, p = 0.482), and creatinine (OR: 0.938, p = 0.135). Diastolic blood pressure was inversely related (OR: 0.994, p < 0.001), while systolic blood pressure was positively associated (OR: 1.012, p < 0.001). In addition, eGFR (OR: 1.002, p = 0.020) and BUN (OR: 1.021, p < 0.001) were also significant predictors.

We further examined the dose-response effect between uric acid levels and the prevalence of KSD across quartiles of uric acid concentration in both male and female participants **(Table 4)**. For males, the prevalence rate of KSD was relatively stable across the uric acid quartiles, with a slight increase in the highest quartile (> 6.3 mg/dL) where the OR was 1.151 (95% CI: 1.009-1.314; p = 0.037) in the age-adjusted model. However, this association was not significant in the multivariable-adjusted model (OR: 1.018, p = 0.800). For females, a stronger positive association was observed, particularly in the higher uric acid quartiles. In the highest quartile, the prevalence was 6.7%, with an age-adjusted OR of 1.782 (95% CI: 1.588-2.001; p < 0.001) and a multivariable-adjusted OR of 1.437 (95% CI: 1.260-1.639; p < 0.001). These findings showed that higher uric acid levels were more strongly associated with an increased risk of KSD in the females than in the males, with significant associations persisting even after adjusting for multiple variables (Table 4).

Because gout is a clinical manifestation of longstanding hyperuricemia and may independently contribute to KSD, we further examined the joint effect of hyperuricemia and gout by stratifying participants into four exposure groups. Among the 118,963 participants, 94,206 without hyperuricemia or gout had a KSD prevalence of 5.4% (5,097 cases), 20,199 with hyperuricemia but no gout had a prevalence of 8.5% (1,717 cases), 1,663 with gout but no hyperuricemia had a prevalence of 17.0% (282 cases), and 2,895 with both hyperuricemia and gout had a prevalence of 15.7% (454 cases) (**Table 5**). Compared with the reference group (no hyperuricemia and no gout), the adjusted odds of KSD were 1.178 (95% CI: 1.107-1.253; p < 0.001) for hyperuricemia without gout, 1.522 (95% CI: 1.325-1.745; p < 0.001) for gout without hyperuricemia, and 1.505 (95% CI: 1.345-1.682; p < 0.001) for both conditions (**Table 5**).

Finally, to investigate whether the association between hyperuricemia and KSD is mediated by gout, we conducted a causal mediation analysis with gout as the mediator. The total effect of hyperuricemia on KSD was statistically significant (Total Effect = 0.01544; 95% CI: 0.01145-0.02000; p < 2*×10⁻¹⁶) **(Table 6)**. The average causal mediation effect (ACME), representing the indirect effect through gout, was estimated at 0.00370 (95% CI: 0.00300-0.00440; p < 2*10⁻¹⁶). The average direct effect (ADE) of hyperuricemia on KSD, independent of gout, remained significant (ADE = 0.01173; 95% CI: 0.00773-0.02000; p < 2*10⁻¹⁶). Notably, the proportion of the total effect mediated by gout was approximately 24% (Proportion Mediated = 0.2399; 95% CI: 0.17656-0.33000).

## Discussion

In the large cohort of 118,963 participants from the TWB in this study, hyperuricemia was shown to be an independent risk factor for kidney stones, with this association remaining significant even after adjusting for multiple confounding variables (OR = 1.155, p < 0.001). The results also revealed significant sex differences, with the risk of kidney stone formation in the female participants with hyperuricemia being significantly higher in those in the highest quartile of uric acid (OR = 1.437, p < 0.001), whereas no significant correlation was found in the males. This sex difference may be related to differences in uric acid metabolism, hormonal changes, and physiological characteristics. This research is the largest study to date in Asia examining the link between hyperuricemia and kidney stones, and it is the first to identify significant sex differences in the impact of hyperuricemia on the risk of kidney stone formation.

Our results highlight the significant association between hyperuricemia and KSD, extending previous findings by Kramer et al. and Pak et al., who also examined the relationship between elevated serum uric acid levels and KSD 29,30. Kramer et al. analyzed a large male cohort of medical professionals, focusing on gout as an indirect indicator of hyperuricemia. Their results showed that gout was a significant independent risk factor for KSD (relative risk = 2.12; 95% CI: 1.22-3.68), suggesting that abnormal uric acid metabolism in gout patients, particularly reduced urine pH, promotes uric acid crystallization 29.

However, their study only included male participants, limiting its applicability to women, and it did not directly evaluate serum uric acid as an independent risk factor, using gout instead as a surrogate indicator. In contrast, our study directly measured serum uric acid levels in a broader population, including both men and women (n = 118,963). As shown in Table 2, we confirmed a strong positive association between hyperuricemia and KSD risk (OR = 1.744; p < 0.001). Even after adjusting for key confounders in multivariate analysis (see Table 3), this association remained significant (OR = 1.155; p < 0.001). By including both male and female participants, our findings emphasize the need for sex-specific considerations when assessing the risk of KSD associated with hyperuricemia, an aspect not addressed in Kramer's study.

Pak et al. investigated the biochemical mechanisms of idiopathic uric acid kidney stones, focusing on the metabolic factors influencing stone formation 30. They found that low urine pH was the primary driver of uric acid stone formation, and that serum uric acid levels in the patients with uric acid stones were significantly higher than in controls (7.1 mg/dL vs. 5.3 mg/dL, p < 0.001). They suggested that hyperuricemia combined with acidic urine increases the risk of uric acid crystallization, and highlighted the key role of impaired ammonium ion production in maintaining a low urinary pH environment. However, their study was limited by a small sample size (only 56 patients) and did not examine sex differences. Our study builds on their findings by investigating the epidemiological relationship between serum uric acid levels and KSD risk in a larger population. Sex-stratified analysis (see Table 4) revealed a significant difference between the female and male participants, and the women in the highest serum uric acid quartile had a significantly increased risk of KSD (OR = 1.437; p < 0.001), while adjusted results for men did not reach statistical significance (OR = 1.018; p = 0.800). This suggests that hyperuricemia may play a more critical role in KSD pathogenesis in women, potentially due to differences in uric acid metabolism or hormonal changes post menopause.

In our combined exposure analysis, participants with hyperuricemia but without gout had a moderately elevated risk of KSD, while those with gout, regardless of serum uric acid status, exhibited the highest risks. Interestingly, the risk estimates for gout alone and for the coexistence of hyperuricemia and gout were comparable, suggesting that once individuals develop gout, the incremental effect of elevated serum uric acid may be limited. Clinically, this is plausible because gout represents a symptomatic manifestation of longstanding hyperuricemia, and the stone risk in these individuals is likely driven not only by hyperuricemia but also by persistently low urinary pH and urate crystal deposition. These findings underscore gout as a strong clinical marker of KSD risk.

To further clarify the relationship, we conducted a causal mediation analysis treating gout as a mediator of the association between hyperuricemia and KSD. We found that approximately 24% of the total effect of hyperuricemia on KSD was mediated through gout, while the majority (76%) was attributable to a direct effect independent of gout. This is consistent with previous studies demonstrating gout as a downstream complication of hyperuricemia that contributes to nephrolithiasis through urate crystal deposition, inflammation, and altered renal function 31-33. However, we also observed a significant direct effect of hyperuricemia on KSD independent of gout, suggesting that other mechanisms, including subclinical crystal formation, oxidative stress, and metabolic disturbances, may also contribute 34,35. Notably, prior epidemiological research has largely focused on gout populations 29, whereas our study provides evidence that elevated serum uric acid levels alone are harmful, even in individuals without clinically apparent gout. These findings underscore the need to consider hyperuricemia management not only for gout prevention but also for reducing kidney stone risk.

Our findings contrast sharply with those of Xu et al., who analyzed a large cohort of over 80,000 Chinese adults and observed a strong dose-response relationship between serum uric acid levels and KSD risk in men, with the risk increasing by 10.7% for each 50 µmol/L increase in serum uric acid above 330 µmol/L 36. However, they did not find a significant association in women. In comparison, our study used a larger, more diverse sample from the TWB including both men and women and adjusting for a broader range of lifestyle factors (such as BMI, smoking, alcohol consumption, hypertension, diabetes, and gout). After comprehensive multivariate adjustments, we found a significant association between elevated serum uric acid levels and KSD risk in women (OR = 1.437; p < 0.001). This suggests that hyperuricemia may have a stronger pathological impact in women, differing from Xu et al.'s findings, and highlighting the need for sex-specific risk assessments.

Deng et al. reported sex differences in the risk of hyperuricemia across different populations, noting a more significant association between hyperuricemia and KSD in men 37. They analyzed a multi-ethnic Chinese cohort, including Han, Yi, and Bai populations, using restricted cubic spline regression to assess the dose-response relationship between serum uric acid levels and KSD risk. Their results showed a significantly higher risk of KSD in men when the serum uric acid exceeded 356 µmol/L, while the threshold for women was 265 µmol/L. However, they did not conduct a thorough sex-stratified analysis or explore sex-specific risk factors. In contrast, we performed comprehensive sex-stratified analysis, and found a significant association between hyperuricemia and KSD in women in the highest serum uric acid quartile (adjusted OR = 1.437, p < 0.001). This novel finding was not addressed in Deng et al.'s study, underscoring the deeper insights provided by our sex-specific analysis.

Previous studies have suggested that the sex difference in the risk of KSD is closely related to the decline in estrogen levels after menopause. Xu et al. (2022) reported that estrogen reduces serum uric acid levels by increasing the renal tubular excretion of uric acid and inhibiting the expressions of stone-forming factors such as urinary calcium and oxalate concentrations 36. However, the protective effect weakens after menopause as estrogen levels drop, leading to a gradual increase in serum uric acid levels. Hak and Choi (2008) also reported increased serum uric acid levels in post-menopausal women 38, and that hyperuricemia was an independent risk factor for kidney stones. Deng et al. (2023) supported this view, showing a significant increase in KSD risk among post-menopausal women, likely due to diminished regulation of uric acid metabolism caused by estrogen deficiency 37.

Despite our findings supporting a positive association between hyperuricemia and kidney stone risk, particularly in women, Narang et al. did not find a significant causal relationship between serum uric acid levels and kidney stone formation in Mendelian randomization analysis 20. Although Mendelian randomization analysis reduces confounding variables and reverse causality, their study had notable limitations, including that they primarily enrolled European participants and lacked Asian participants, in whom hyperuricemia is more prevalent. In addition, they did not account for crucial variables such as urine pH and stone type, potentially underestimating the role of uric acid in specific stone formation.

In contrast to our findings and the traditional view that high uric acid levels increase stone risk, Ferraro and Curhan (2017) found a negative correlation between uric acid excretion and kidney stone risk 21. However, their study had several limitations, including a predominantly American healthcare professional sample, making it less applicable to Asian populations. Moreover, they focused on urinary uric acid excretion rather than directly measuring serum uric acid levels, missing the broader impact of hyperuricemia on kidney stone risk. The larger representative sample and comprehensive analysis in our study provides stronger evidence supporting hyperuricemia as an independent risk factor for kidney stone formation, particularly in women with elevated serum uric acid levels. The difference between our study and Curhan et al.'s underscores the importance of population-specific analyses.

Hyperuricemia promotes kidney stone formation through multiple mechanisms. The deposition of uric acid crystals in the kidneys can induce oxidative stress, inflammation, and endothelial dysfunction, leading to kidney damage and facilitating stone formation 39. One key mechanism is the activation of the NLRP3 inflammasome, which has been shown to exacerbate inflammatory responses and trigger pyroptosis in renal tubular cells, worsening kidney injury and promoting crystal aggregation 40. In addition, metabolic imbalances such as purine and amino acid metabolism abnormalities further impair renal function, increasing susceptibility to stone formation 41. Hyperuricemia has also been shown to induce epithelial-mesenchymal transition and renal fibrosis, both of which are critical factors in the pathophysiology of kidney stone formation 42. These interrelated pathways highlight the multifaceted role of hyperuricemia in the development of kidney stones.

### Limitations

This study has several limitations that warrant consideration. A major limitation is the lack of data on kidney stone composition. As different stone types arise from distinct pathophysiological mechanisms, it is unclear whether the observed association with hyperuricemia in women reflects calcium oxalate, calcium phosphate, uric acid, ammonium urate, brushite, cystine, or other subtypes. Future studies incorporating stone composition and urinary biochemistry are warranted to elucidate the underlying mechanisms. Other limitations include the following. First, the cross-sectional design precludes the establishment of causal relationships between hyperuricemia and kidney stones, limiting our ability to determine temporal relationships. Second, the reliance on self-reported kidney stone diagnoses may have introduced recall bias and potentially led to cases being misclassified, possibly affecting the accuracy of our results. Third, the study population was drawn exclusively from the TWB and may not be fully representative of the broader Taiwanese population or other ethnic groups, potentially limiting the generalizability of our findings. Fourth, the lack of longitudinal follow-up data restricted our ability to assess the long-term impact of hyperuricemia on kidney stone formation and recurrence. Fifth, the number of KSD cases (n = 7,550) exceeded that of CKD cases (n = 1,892). Although KSD and CKD are related, they are distinct entities, and most stone formers, particularly in a relatively healthy cohort such as the TWB, do not develop CKD. Prior population-based studies similarly indicate that while KSD increases the relative risk of CKD, the absolute progression rate is modest 43-45. Accordingly, the higher prevalence of KSD is expected, and our analysis should be interpreted as addressing hyperuricemia and KSD in the general population rather than in a CKD subgroup. Lastly, while we adjusted for numerous covariates, residual confounding from unmeasured factors such as dietary habits, fluid intake, and certain medications cannot be ruled out. These limitations underscore the need for future prospective studies with more diverse populations and comprehensive assessments of potential confounders to further elucidate the relationship between hyperuricemia and KSD.

## Conclusion

The results of this large Taiwanese cohort study showed that hyperuricemia was an independent risk factor for KSD, with a notably stronger association in women compared to men. The significant sex difference suggests the potential influence of hormonal factors such as post-menopausal decline in estrogen on uric acid metabolism and stone formation. Our causal mediation analysis further revealed that approximately one-quarter of the association between hyperuricemia and KSD was mediated by gout, while the direct effect of hyperuricemia remained significant, indicating additional mechanisms beyond clinically apparent gout. These findings emphasize the need for sex-specific risk assessments and management strategies in patients with elevated serum uric acid levels. Given the rising incidence of KSD and the high prevalence of hyperuricemia, regular monitoring of uric acid levels and targeted interventions could be effective in reducing KSD risk, particularly in high-risk groups. Future longitudinal studies are warranted to explore the causal pathways and validate these associations across diverse populations.

## Figures and Tables

**Table 1:** Basic characteristics of the participants

		Hyperuricemia exposure		
**Characteristics**	**Total** **=118,963**	**No exposure95,869**	**Exposure 23,094**	**p value**
**Demographic data**				
Age, yr	50 ± 11	50 ± 11	51 ± 11	<0.001
Women n (%)	76,250(64.1)	65,890(68.7)	10,360(44.9)	<0.001
Body mass index, kg/m^2^	24.22 ± 3.78	23.67 ± 3.53	26.50 ± 3.93	<0.001
Smoking status, ever, n (%)	32,425(27.3)	23,728(24.8)	8,697(37.7)	<0.001
Alcohol status, yes, n (%)	10,137(8.5)	6,921(7.2)	3,216(13.9)	<0.001
Physical activity, yes, n (%)	48,325 (40.6)	38,660 (40.3)	9,665 (41.9)	<0.001
Systolic blood pressure, mmHg	120.52 ± 18.67	118.93 ± 18.36	127.15 ± 18.46	<0.001
Diastolic blood pressure, mmHg	73.83 ± 11.40	72.74 ± 11.11	78.38 ± 11.47	<0.001
**Comorbidity**				
Hypertension, n (%)	14,581(12.3)	9,750(10.2)	4,831(20.9)	<0.001
Diabetes mellitus, n (%)	6,113(5.1)	4,655(4.9)	1,458(6.3)	<0.001
Metabolic syndrome	35,246(29.6)	24,033(25.1)	11,213(48.6)	<0.001
CKD, n (%)	1,892(1.6)	765(0.8)	1,127(4.9)	<0.001
Gout, n (%)	4,558(3.8)	1,663(1.7)	2,895(12.5)	<0.001
**Lab data**				
HbA1c, % (%)	5.8±0.8	5.7 ± 0.8	5.9 ± 0.7	<0.001
eGFR, ml/min/1.73 m^2^	103±24	106±24	91±21	<0.001
BUN, mg/dL	13.11±3.92	12.82 ± 3.60	14.32 ± 4.84	<0.001
Creatinine, mg/dL	0.72±0.31	0.69 ± 0.26	0.85 ± 0.44	<0.001
Uric acid	5.43 ± 1.43	4.93 ± 0.99	7.50 ± 1.02	<0.001
**Kidney stone disease, n (%)**	7,550 (6.3%)	5,379 (5.6%)	2,171 (9.4%)	<0.001

CKD = chornic kidney disease; HbA1c = Hemoglobin A1c; eGFR = estimated glomerular filtration rate; BUN = blood urea nitrogen

**Table 2:** The association between hyperuricemia and kidney stone disease based on univariate binary logistic regression analysis

Characteristic	Odds ratio (95% CI)	P value
Age (per 1 year)	1.036(1.034-1.039)	<0.001
Women (*vs.* men)	2.914(2.778-3.057)	<0.001
Body mass index (per 1 kg/m^2^)	1.075(1.069-1.081)	<0.001
Smoking status, ever (*vs.* never)	1.973(1.881-2.069)	<0.001
Alcohol status, ever (*vs.* never)	1.722(1.606-1.847)	<0.001
Regular physical activity, yes (*vs.* no)	1.236(1.179-1.295)	<0.001
Systolic blood pressure (per 1 mmHg)	1.017(1.015-1.018)	<0.001
Diastolic blood pressure (per 1 mmHg)	1.029(1.027-1.031)	<0.001
Hypertension, yes (*vs.* no)	2.776(2.629-2.932)	<0.001
Diabetes mellitus, yes (*vs.* no)	2.148(1.963-2.307)	<0.001
Metabolic syndrome, yes (*vs.* no)	2.004(1.911-2.100)	<0.001
CKD, yes (*vs.* no)	2.958(2.613-3.349)	<0.001
Gout, yes (*vs.* no)	3.042(2.801-3.304)	<0.001
HbA1c (per 1%)	1.216(1.190-1.242)	<0.001
eGFR (per 1 ml/min/1.73 m^2^)	0.986(0.985-0.987)	<0.001
BUN (per 1 mg/dL)	1.064(1.059-1.069)	<0.001
Creatinine (per 1 mg/dL)	1.759(1.645-1.880)	<0.001
Hyperuricemia, yes (*vs.* no)	1.744(1.656-1.837)	<0.001

CKD = chornic kidney disease; HbA1c = Hemoglobin A1c; eGFR = estimated glomerular filtration rate; BUN = blood urea nitrogen; CI = confidence interval

**Table 3:** The association between hyperuricemia and kidney stone disease based on multivariate binary logistic regression analysis

Characteristic	Odds ratio (95% CI)	P value
Age (per 1 year)	1.030 (1.027 - 1.033)	<0.001
Women (*vs.* men)	2.332 (2.190 - 2.483)	<0.001
Body mass index (per 1 kg/m^2^)	1.028 (1.020 -1.035)	<0.001
Smoking status, ever (*vs.* never)	1.118 (1.055 - 1.186)	<0.001
Alcohol status, ever (*vs.* never)	0.940 (0.872 - 1.015)	0.113
Regular physical activity, yes (*vs.* no)	0.961 (0.913 - 1.011)	0.124
Systolic blood pressure (per 1 mmHg)	1.012 (1.009 - 1.015)	<0.001
Diastolic blood pressure (per 1 mmHg)	0.994 (0.992 - 0.996)	<0.001
Hypertension, yes (*vs.* no)	1.611 (1.511 - 1.717)	<0.001
Diabetes mellitus, yes (*vs.* no)	1.214 (1.098 - 1.342)	<0.001
Metabolic syndrome, yes (*vs.* no)	1.091 (1.026 - 1.161)	0.005
CKD, yes (*vs.* no)	1.209 (1.038 - 1.407)	0.015
Gout, yes (*vs.* no)	1.370 (1.253 -1.499)	<0.001
HbA1c (per 1%)	0.989 (0.957 - 1.021)	0.482
eGFR (per 1 ml/min/1.73 m^2^)	1.002 (1.000 - 1.003)	0.020
BUN (per 1 mg/dL)	1.021 (1.014 -1.028)	<0.001
Creatinine (per 1 mg/dL)	0.938 (0.862- 1.020)	0.135
Hyperuricemia, yes (*vs.* no)	1.155 (1.089 - 1.224)	<0.001

* Adjusted for age, sex, body mass index, smoking, alcohol, regular physical activity, systolic blood pressure, diastolic blood pressure, past history of hypertension, diabetes mellitus, metabolic syndrome, chronic kidney disease, gout, Hemoglobin A1c, eGFR, blood urea nitrogen, and creatinine. CKD = chornic kidney disease; HbA1c = Hemoglobin A1c; eGFR = estimated glomerular filtration rate; BUN = blood urea nitrogen; CI = confidence interval

**Table 4:** Odds ratios for kidney stone disease stratified by uric acid quartile and sex

Uric Acid Quartile (mg/dL)	Participants (KSD Cases, Prevalence)	Age-Adjusted OR (95% CI)	P value	Multivariable-Adjusted OR (95% CI)	P value
**Male Participants (n = 42,713)**					
1st (<4.4)	2,567 (280, 10.9%)	Ref	-	Ref	-
2nd (4.4-5.3)	6,345 (623, 9.8%)	0.928 (0.799-1.078)	0.329	0.975 (0.837-1.134)	0.740
3rd (5.3-6.3)	12,606 (1,293, 10.3%)	1.014 (0.884-1.164)	0.839	1.042 (0.906-1.199)	0.564
4th (>6.3)	21,195 (2,355, 11.1%)	1.151 (1.009-1.314)	0.037	1.018 (0.887-1.168)	0.800
**Female Participants (n = 76,250)**					
1st (<4.4)	28,987 (914, 3.2%)	Ref	Ref	Ref	Ref
2nd (4.4-5.3)	24,655 (872, 3.5%)	1.037 (0.943-1.140)	0.452	1.004 (0.912-1.106)	0.932
3rd (5.3-6.3)	15,451 (734, 4.8%)	1.321 (1.195-1.461)	<0.001	1.205 (1.083-1.341)	0.001
4th (>6.3)	7,157 (479, 6.7%)	1.782 (1.588-2.001)	<0.001	1.437 (1.260-1.639)	<0.001

* Multivariable-Adjusted for or age, body mass index, smoking, alcohol, regular physical activity, systolic blood pressure, diastolic blood pressure, past history of hypertension, diabetes mellitus, metabolic syndrome, chronic kidney disease, gout, Hemoglobin A1c, estimated glomerular filtration rate, blood urea nitrogen, and creatinine. KSD = kidney stone disease; OR = odds ratio; CI = confidence interval

**Table 5:** Association of combined hyperuricemia and gout status with kidney stone disease.

Exposure group	n	KSD cases (%)	Prevalence (%)	OR (95% CI), Crude	p value	OR (95% CI), Adjusted	p value
No HU, No Gout	94,206	5,097 (5.4)	5.4	Ref	-	Ref	-
HU, No Gout	20,199	1,717 (8.5)	8.5	1.624 (1.534-1.719)	<0.001	1.178 (1.107-1.253)	<0.001
No HU, Gout	1,663	282 (17.0)	17.0	3.570 (3.126-4.063)	<0.001	1.522 (1.325-1.745)	<0.001
HU, Gout	2,895	454 (15.7)	15.7	3.252 (2.927-3.605)	<0.001	1.505 (1.345-1.682)	<0.001

* Multivariable-Adjusted for or age, body mass index, smoking, alcohol, regular physical activity, systolic blood pressure, diastolic blood pressure, past history of hypertension, diabetes mellitus, metabolic syndrome, chronic kidney disease, gout, Hemoglobin A1c, estimated glomerular filtration rate, blood urea nitrogen, and creatinine. KSD = kidney stone disease; OR = odds ratio; CI = confidence interval; HU = Hyperuricemia

**Table 6:** Causal mediation analysis of the association between hyperuricemia, gout, and kidney stone disease

Parameter	Estimate	95% CI Lower	95% CI Upper	P value
ACME (control)	0.00345	0.00277	0.00400	<2*10^-16^
ACME (treated)	0.00396	0.00321	0.00400	<2*10^-16^
ADE (control)	0.01148	0.00756	0.02000	<2*10^-16^
ADE (treated)	0.01199	0.00791	0.02000	<2*10^-16^
Total Effect	0.01544	0.01145	0.02000	<2*10^-16^
Prop. Mediated (control)	0.22349	0.16180	0.32000	<2*10^-16^
Prop. Mediated (treated)	0.25631	0.19128	0.35000	<2*10^-16^
ACME (average)	0.00370	0.00300	0.00400	<2*10^-16^
ADE (average)	0.01173	0.00773	0.02000	<2*10^-16^
Prop. Mediated (average)	0.23990	0.17656	0.33000	<2*10^-16^

ACME = average causal mediation effect; ADE = average direct effect; Prop. Mediated = proportion of total effect mediated; CI = confidence interval
